# Multidisciplinary management of concurrent small cell lung cancer and hepatocellular carcinoma: A rare case report with long-term survival

**DOI:** 10.1097/MD.0000000000044341

**Published:** 2025-09-12

**Authors:** Ying Li, Xu Zhang, Yi-Qian Wang, Hai-Chen Lv, Shi-Lei Zhao, Zhi-Yong Li, Jun-Ying Wang, Yi Zhao

**Affiliations:** a Department of Oncology, The First Affiliated Hospital of Dalian Medical University, Dalian Medical University, Dalian, China; b Department of Thoracic Surgery, The Second Hospital of Dalian Medical University Cardiovascular Hospital, Dalian, China; c Department of Radiotherapy, The First Affiliated Hospital of Dalian Medical University, Dalian Medical University, Dalian, China; d Department of Cardiology, The First Affiliated Hospital of Dalian Medical University, Dalian Medical University, Dalian, China; e Department of Thoracic Surgery, The First Affiliated Hospital of Dalian Medical University, Dalian Medical University, Dalian, China; f Department of Radiology, The First Affiliated Hospital of Dalian Medical University, Dalian Medical University, Dalian, China; g Department of Pathology, The First Affiliated Hospital of Dalian Medical University, Dalian Medical University, Dalian, China.

**Keywords:** immunotherapy, multiple primary neoplasms, patient care team, small cell lung carcinoma, survival analysis

## Abstract

**Rationale:**

Coexisting small cell lung cancer (SCLC) and hepatocellular carcinoma (HCC) is extraordinarily uncommon; in elderly patients with cardiac dysfunction it demands highly individualized, cardio-conscious care.

**Patient concerns:**

A 72-year-old man experienced an acute myocardial infarction in September 2019 and underwent emergency percutaneous coronary intervention. He had well-controlled hypertension and diabetes but no viral hepatitis history.

**Diagnoses:**

Follow-up contrast computed tomography (December 2019) showed a 1.4 cm left-upper-lobe nodule and an 8 cm hepatic mass. Video-assisted biopsy confirmed stage IIIA SCLC (pT1N2M0). Right-hepatectomy pathology established primary HCC. Baseline echocardiography revealed New York Heart Association class III heart failure with left-ventricular ejection fraction of 40%.

**Interventions:**

The multidisciplinary team performed thoracoscopic lobectomy with mediastinal dissection (January 2020) followed by right hepatectomy (November 2020). Adjuvant therapy was withheld because of severe cardiac compromise. On SCLC progression, the patient received 4 cycles of carboplatin–etoposide, thoracic conformal radiotherapy, and atezolizumab maintenance. Concurrent cardio-oncology management – guideline-directed heart failure medication, serial echocardiography, and endocrinologic treatment of immune-related subclinical hypothyroidism – mitigated therapy-related toxicity.

**Outcomes:**

The patient achieved a durable partial response lasting >18 months. Left ventricular ejection fraction remained ≥40% after temporary decline, and immune adverse effects were controlled. Overall survival reached 39 months; he ultimately died of COVID-19 pneumonia in January 2023.

**Lessons:**

Meticulous multidisciplinary team coordination can balance oncologic efficacy and cardioprotection, enabling meaningful survival for patients with simultaneous SCLC and HCC complicated by severe cardiac dysfunction.

## 1. Introduction

Multiple primary tumors refer to the simultaneous occurrence of 2 or more primary tumors in a single individual, either at disparate anatomic sites or within the same site.^[1]^ Although the occurrence of multiple primary tumors is infrequent, it remains a plausible scenario. As elucidated in a recent investigation, the incidence of multiple primary tumors is on the rise, with an estimated prevalence of approximately 1 in 20 individuals afflicted by cancer concurrently.^[1,2]^ The etiological underpinnings behind the development of 2 distinct primary cancers during an individual’s lifetime are intricate, encompassing genetic factors, behavioral influences, and previous cancer treatment.^[3]^ The diagnosis and management of patients with multiple primary tumors can be challenging, and it requires a multidisciplinary approach.^[4]^

Small cell lung cancer (SCLC) and hepatocellular carcinoma (HCC) are both aggressive malignancies with distinct anatomical origins and therapeutic approaches. Sidhom et al reported a very rare case of primary HCC and non-small-cell lung adenocarcinoma occurring concurrently.^[5]^ Here, we present a case of a patient who presented with compromised cardiac function and was subsequently diagnosed with both SCLC and HCC. Following comprehensive management by a multidisciplinary team (MDT), the patient achieved a remarkable 3-year overall survival.

## 2. Case presentation

A 72-year-old man with well-controlled hypertension and type 2 diabetes was admitted in September 2019 for an ST-elevation myocardial infarction treated by percutaneous coronary intervention. Baseline echocardiography showed a left ventricular ejection fraction (LVEF) of 40%. He had never smoked and had no family or viral hepatitis history.

**Figure 1.**
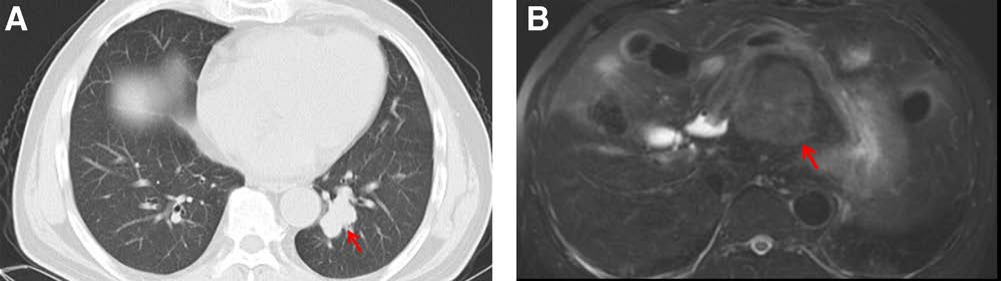
Baseline cross-sectional imaging (December 2019). (A) Chest CT showed a lobulated soft tissue nodule in the left lower lobe (red arrow; 3.1 cm × 2.3 cm). (B) MRI demonstrated a solitary hyperintense mass in the left hepatic lobe (red arrow; 5.9 cm × 4.7 cm). CT = computed tomography, MRI = magnetic resonance imaging.

**Figure 2.**
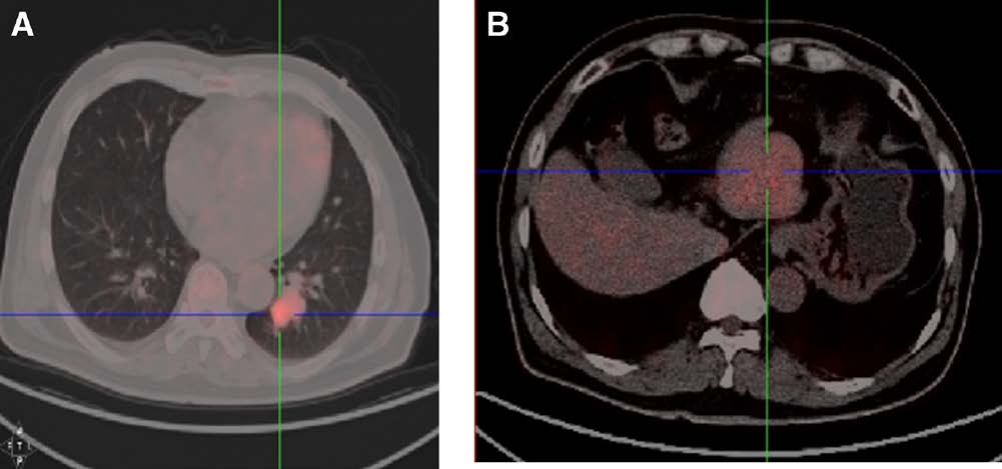
Baseline 18F-FDG PET/CT revealed an intense FDG uptake (A) in the pulmonary lesion (SUV_max_ 8.2; left) and (B) in the left-lobe hepatic mass (SUV_max_ 5.2; right), supporting dual primary malignancies. CT = computed tomography, FDG = fluorodeoxyglucose, PET = positron emission tomography, SUV = standard uptake value.

At routine follow-up in December 2019, contrast chest computed tomography (CT) identified a lobulated 3.1 cm × 2.3 cm nodule in the left lower lobe (Fig. 1A). Concomitant abdominal CT/magnetic resonance imaging revealed a solitary 5.9 cm × 4.7 cm mass in the left hepatic lobe (Fig. 1B). 18F-fluorodeoxyglucose positron emission tomography (PET)/CT demonstrated intense uptake in both foci (lung standard uptake value_max_ 8.2; liver standard uptake value_max_ 5.2; Fig. 2). Serum B-type natriuretic peptide was 160 pg/mL and pro-gastrin-releasing peptide (Pro-GRP) 426 pg/mL; other tumor markers (NSE, CEA, SCC, C21-1, and alpha-fetoprotein [AFP]) were normal. The imaging suggested stage I lung cancer (cT1N0M0) and radiologic stage IIIA HCC (cT3N0M0), but the patient declined liver biopsy.

**Figure 3.**
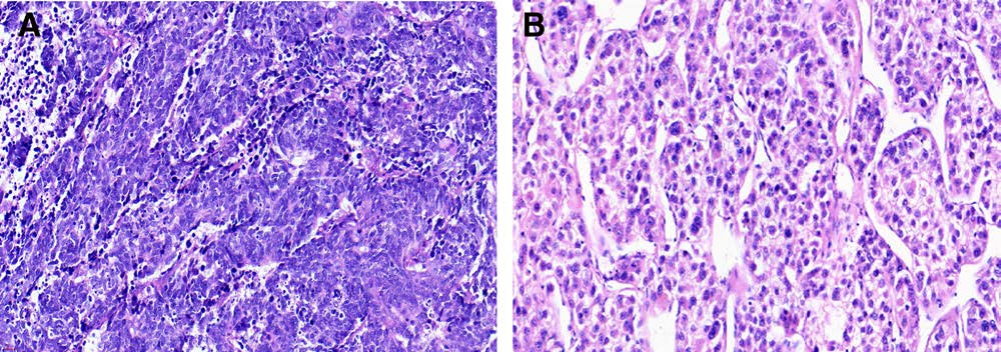
Surgical pathology of dual primaries. (A) Small-cell lung cancer resected in January 2020: hematoxylin–eosin (H&E; ×400) showed densely packed small cells with scant cytoplasm. (B) Moderately differentiated hepatocellular carcinoma excised in November 2020: H&E showed trabecular architecture with cytoplasmic eosinophilia (×400). H&E = hematoxylin–eosin.

**Figure 4.**
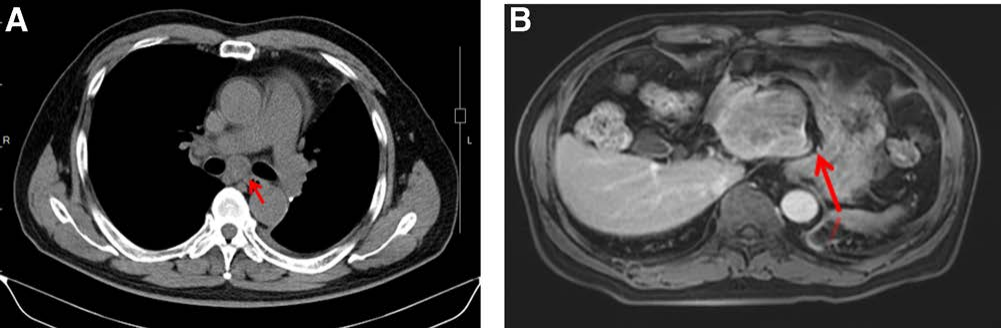
First radiological progression (May 2020). (A) Chest CT identified enlarged mediastinal lymph nodes (red arrow; 2.6 cm). (B) Abdominal MRI showed interval growth of the left-lobe mass to 7.3 cm × 6.0 cm (red arrow). CT = computed tomography, MRI = magnetic resonance imaging.

Video-assisted thoracoscopic lobectomy in January 2020 confirmed small-cell lung cancer with N2 involvement (pT1N2M0; Fig. 3A). Adjuvant chemotherapy was deferred because of cardiac risk. In May 2020 surveillance CT showed multiple mediastinal nodes (largest 2.6 cm) and enlargement of the hepatic lesion to 7.3 cm × 6.0 cm (Fig. 4A, B); Pro-GRP had risen to 2077 pg/mL and AFP to 38.6 IU/mL. Four cycles of etoposide and carboplatin (EC; June–August 2020) produced a partial response, but precipitated symptomatic heart failure (LVEF 30%, B-type natriuretic peptide 1660 pg/mL).

Despite systemic control, the liver mass progressed and AFP climbed to 88 IU/mL. Laparoscopic left hepatectomy (November 2020) yielded a moderately differentiated HCC (Fig. 3B). Three months later (January 2021) PET/CT revealed new fluorodeoxyglucose-avid mediastinal nodes; Pro-GRP had risen to 2253 pg/mL. Involved-field mediastinal radiotherapy followed by prophylactic cranial irradiation produced another partial response.

In May 2021 CT showed recurrent mediastinal adenopathy, moderate pericardial effusion and multiple sub-centimeter liver nodules; PET/CT confirmed diffuse metastatic SCLC. Tumor markers were Pro-GRP 2720 pg/mL and NSE 72 ng/mL. On the basis of IMpower133 data,^[6]^ he received atezolizumab plus EC for 4 cycles, then 18 cycles of maintenance atezolizumab, achieving durable control and only subclinical hypothyroidism.

**Figure 5.**
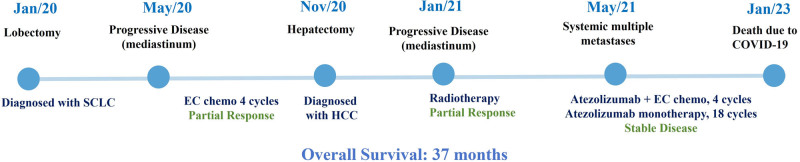
The flowchart of the patient’s diagnosis and treatment course. COVID-19 = Coronavirus disease 2019, EC = etoposide and carboplatin, HCC = hepatocellular carcinoma, SCLC = small cell lung cancer.

He died of COVID-19–related cardiorespiratory failure in January 2023. Figure 5 shows the timeline and flowchart of the patient’s diagnosis and treatment course, with an overall survival of 37 months from first oncologic imaging.

## 3. Discussion

In this case report, we present a unique case of concurrent SCLC and HCC in an elderly patient. Despite the patient’s dual diagnosis of highly malignant tumors and preexisting poor cardiac function (New York Heart Association class III), the patient derived significant benefits from the MDT approach, ultimately achieving an overall survival of over 3 years.

SCLC is characterized by a notable propensity for early metastasis and an exceedingly unfavorable prognosis.^[7]^ Systemic chemotherapy serves as the cornerstone of therapeutic management for SCLC across all stages. Currently, the preferred chemotherapeutic strategy for SCLC involves combinations of platinum-based agents (cisplatin or carboplatin) and topoisomerase inhibitors (etoposide or irinotecan). These combinations have shown notable efficacy and have been extensively studied for safety, whether used alone or combined with immunotherapy or radiotherapy or surgery.^[8]^ This systemic approach for SCLC stands in contrast to the management of non-SCLC, where targeted therapies against specific driver mutations, such as anaplastic lymphoma kinase, have become a cornerstone of treatment for eligible patients.^[9]^ However, due to the diverse toxicities of these cytostatic agents, treatment should be tailored to the individual patient, taking into consideration their oval condition, comorbidity, and individual preferences.^[10]^

Considering the patient’s advanced age and compromised cardiac function, the MDT opted not to administer adjuvant therapy following the patient’s lung surgery. Following the onset of SCLC metastasis, the patient was recommended to undergo 4 cycles of chemotherapy using the EC regimen. This regimen proved effective, as the patient demonstrated PR. Cisplatin and carboplatin are both platinum-based chemotherapeutic agents, but they have different molecular structures and mechanisms of action.^[11]^ It is reported that carboplatin has a more favorable toxicity profile than cisplatin, including lower heart toxicity. These differences may contribute to the varying toxicities observed, including heart toxicity.^[12]^

According to the Chinese expert consensus,^[13,14]^ the treatment of elderly patients with lung cancer should be individualized based on their overall health status, comorbidities, and treatment goals. The consensus recommends that the use of chemotherapy, immunotherapy, and radiotherapy should be carefully evaluated in patients with poor heart function, and that the potential benefits and risks of treatment should be weighed. In addition, a study on the risk factors for postoperative arrhythmia in elderly lung cancer patients found that elderly patients with lung cancer are prone to arrhythmia due to the trauma of surgery, the effects of anesthesia on the heart, pain, bleeding, hypoxia, and electrolyte and acid–base imbalances. Therefore, it is important to consider the patient’s overall condition and the potential benefits and risks of treatment when making treatment decisions for lung cancer patients with poor heart function. A multidisciplinary approach involving oncologists, cardiologists, and other specialists may be necessary to optimize patient outcomes.

The management of concurrent SCLC and primary liver cancer requires a comprehensive and individualized approach, considering tumor characteristics, patient comorbidities, and functional status. The MDT plays a pivotal role in guiding treatment decisions and optimizing therapeutic strategies.

Based on the search results, some drugs used in the treatment of primary liver cancer have been associated with cardiac toxicity and should be used with caution in patients with poor heart function. Sorafenib, a tyrosine kinase inhibitor, has been reported to cause heart failure complicated by cardiogenic shock in some patients with advanced stage HCC.^[15]^ Trastuzumab, a monoclonal antibody used to treat HER-2 positive breast cancer, has also been associated with a decrease in LVEF and heart failure.^[16]^ Anthracyclines, a class of chemotherapy drugs used to treat various types of cancer, including liver cancer, are known to cause cardiotoxicity, which can be life-threatening.^[17]^ However, it should be noted that not all patients who receive these drugs will experience cardiac toxicity, and the risk and severity of toxicity may vary depending on the patient’s individual characteristics and the specific drug regimen used. In addition, other molecularly targeted antiangiogenic agents used in the treatment of HCC, such as regorafenib and lenvatinib, have been associated with cardiovascular effects, including hypertension and arterial thromboembolism.

## 4. Conclusion

Therefore, it is important to carefully evaluate the potential benefits and risks of treatment and to monitor the patient’s heart function closely when using these drugs in patients with poor heart function. This case report highlights the importance of MDT approach in managing complex cases.

## Acknowledgments

The relevant authors are all members of the multidisciplinary tumor treatment team. Zhi-Yong Li is a radiologist and provided the current imaging data. We thank for Dr. Bin Qiao for editing this manuscript.

## Author contributions

**Conceptualization:** Ying Li, Yi Zhao.

**Formal analysis:** Yi-Qian Wang, Hai-Chen Lv, Shi-Lei Zhao.

**Funding acquisition:** Yi Zhao.

**Investigation:** Ying Li, Xu Zhang, Yi Zhao.

**Methodology:** Ying Li, Zhi-Yong Li, Jun-Ying Wang.

**Resources:** Yi Zhao.

**Validation:** Ying Li, Xu Zhang, Yi Zhao.

**Visualization:** Yi-Qian Wang.

**Writing – original draft:** Ying Li, Xu Zhang, Yi-Qian Wang, Yi Zhao.
